# Treatment outcome of acute coronary syndrome patients admitted to Ayder Comprehensive Specialized Hospital, Mekelle, Ethiopia; A retrospective cross-sectional study

**DOI:** 10.1371/journal.pone.0228953

**Published:** 2020-02-13

**Authors:** Desilu Mahari Desta, Teshome Nedi, Abraha Hailu, Tesfay Mehari Atey, Afewerki Gebremeskel Tsadik, Solomon Weldegebriel Asgedom, Gebremicheal Gebereslassie Kasahun, Eskinder Ayalew

**Affiliations:** 1 Clinical Pharmacy Unit, School of Pharmacy, College of Health Sciences, Mekelle University, Mekelle, Tigray, Ethiopia; 2 Department of Pharmacology and Clinical Pharmacy, School of Pharmacy, College of Health Sciences, Addis Ababa University, Addis Ababa, Ethiopia; 3 Department of Internal Medicine, School of Medicine, College of Health Sciences, Mekelle University, Mekelle, Tigray, Ethiopia; 4 Department of Pharmacy, College of Health Sciences, Aksum University, Aksum, Tigray, Ethiopia; Azienda Ospedaliero Universitaria Careggi, ITALY

## Abstract

**Background:**

Acute coronary syndrome (ACS) is increasingly becoming a common cause of cardiovascular mortality in developing countries. Even though, there is an introduction of limited percutaneous coronary intervention and thrombolytic therapies, in-hospital mortality due to ACS still remains high in sub-Saharan countries.

**Objective:**

The aim of the study was to assess treatment outcome of ACS patients admitted to Ayder Comprehensive Specialized Hospital, Mekelle, Ethiopia.

**Methods:**

A retrospective cross-sectional study was done by collecting data from patients’ medical records using a data abstraction tool. Data were analyzed using logistic regression to determine crude and adjusted odds ratio. At 95% confidence interval, *p-*value<0.05 was considered as statistically significant.

**Results:**

Of the total 151 patients, in-hospital mortality was found to be 24.5%, and hypertension was the most frequent (46.4%) risk factor of ACS. Concerning the management practice, catheterization and primary percutaneous coronary intervention were done in 27.1%, and 3.9% respectively. Additionally, in emergency setting loading dose of aspirin and clopidogrel were used in about 63.8% and 62.8%, respectively. The other frequently used medications were beta-blockers (86.9%), angiotensin converting enzymes/angiotensin receptor blockers (84.1%) and statins (84.1%). Streptokinase was administered in 6.3% of patients with ST-elevated myocardial infarction and heparins in 78.1% of them. The commonly prescribed discharge medications were aspirin (98.2%), statins (94.7%) and clopidogrel (92%). Non-use of beta-blockers (*p* = 0.014), in-hospital complication of cardiogenic shock (*p* = 0.001) and left ventricular ejection fraction of ≤ 30% (*p* = 0.032) were independent predictors of in-hospital mortality.

**Conclusion:**

The proportion of in-hospital mortality due to ACS was found to be high. Therefore, timely evidence based therapy should be implemented in the setup.

## Background

ACS is a disease of the coronary artery caused due to narrowing or blockage of the coronary artery lumen [1–3]. The narrowing or blockage of the artery causes myocardial cell death due to decreased oxygen supply which is characterized in the form of unstable angina (UA), non-ST-segment elevation myocardial infarction (NSTEMI) and ST-segment elevation myocardial infarction (STEMI) [4]. UA and NSTEMI are similar in pathophysiology and clinical presentations; but their difference is elevation of myocardial necrosis biomarkers in NSTEMI [5].

According to World Health Organization (WHO) prediction, cardiovascular disease (CVD) will continue to be the leading cause of mortality globally up to 2030 [6]. Based on the report of Heart Disease and Stroke Statistics 2018; the global prevalence of ischemic heart disease (IHD) was estimated about 110.6 million, where males were more commonly affected than females and with case fatality rate of 8.9 million [7].ACS is a leading cause of mortality and morbidity, accounts for 50% of all CVD deaths and more than 2.5 million hospitalizations worldwide each year [8].

A prospective study was conducted in Africa, Sub-Saharan countries with total of 111(5.1%) ACS patients comprising of 56% with STEMI and 44% of them NSTEMI/UA. The study claimed that in-hospital mortality was about 6%‒10% in the setting [9]. Moreover, another prospective study in Sub-Saharan Africa population recruited 425 patients, of which 13.5% was the prevalence of ACS. About 71.5% of the total had final diagnosis of STEMI type and 28.5% of them were NSTEMI. The in-hospital mortality was reported to be 10% [10].

Currently, ACS is becoming highly prevalent and poorprognosis CVD in Ethiopia. A cross-sectional study conducted by Yedeta and his colleagues’ on spectrum of cardiovascular diseases in six main referral hospitals of Ethiopia reported that out of 6275 CVD patients, 995 of them were in Ayder Comprehensive Specialized Hospital (ACSH), Mekelle. From the six referral hospitals, IHD accounted 9.6% of CVD [11].

The treatment outcome of ACS was observed poor in Ethiopia, in which patient death and in-hospital complications are increasing. A retrospective cross-sectional study carried out in Tikur Anbessa Specialized Hospital, Ethiopia, from 1981 to 1986 revealed that out of 23 patients with AMI in-hospital mortality was 29.4% [12]. Similarly, another study from Tikur Anbessa Specialized Hospital conducted on the chart review of patients admitted from January 1, 2012 to December 31, 2014 in 124 patients reported that in-hospital mortality was 27.4%. The study claimed also heart failure (16.1%), cardiogenic shock (11.3%) and major arrhythmia (8.1%) were common in-hospital complications [13].

Poor treatment outcome of ACS patients could be due to lack of adequate and/or timely use of evidence-based medical and non-medical therapy utilization which in turn progresses into complications, and mortality [14]. The fact that evidence-based invasive and non-invasive management prevents ACS associated morbidity and mortality [15]. Therefore, Patients’ with ACS require intensive treatment and diagnostic evaluation for greatly improved treatment outcome as well as prolonged survival and better quality of life [14].

In the study setting, little is known about the incidence of ACS admissions and patients’ prognosis. Therefore, the study will contribute a lot on improving the quality of healthcare for patients with ACS by identifying gaps and directing potential solutions. The study assessed risk factors, management practice, treatment outcome and predictors of in-hospital mortality for ACS.

## Methods

A retrospective cross-sectional study was conducted on medical records of patients admitted from August 1, 2013‒July 31, 2018 in Ayder Comprehensive Specialized Hospital (ACSH), found in Mekelle, the capital city of Tigray regional state, 783 Kilometers away from Addis Ababa, Ethiopia. About 173 medical records were found with the diagnosis of ACS within the study period, in which 14 of them were excluded, whereby eight of them were discharged against medical advice and six patients had incomplete medical record information. Furthermore, 159 patients were found eligible for the study and then eight (5%) of them were used for pre-test, adjustment and modification of the data abstraction tool was made accordingly. Finally, 151 patients’ records were used in the actual study.

Health management information system (HMIS) patient registration book was employed for accessing the card numbers of ACS patients admitted from August 1, 2013‒July 31, 2018. Complete records patients admission and discharge of HMIS was found starting from August 1, 2013. Then, patient charts were retrieved and collected from record and documentation office. Data were collected by developing a data abstraction format which was prepared using global registry of acute coronary events (GRACE) as well as incorporating studies from India and European countries [16–21]. Four trained clinical pharmacists who had good experience in the clinical practice were recruited for data collection. Data were collected between July 20, 2018‒August 25, 2018. During the data collection process, the completeness and consistency of the data were checked daily and data collection was supervised.

Data were entered and analyzed using Statistical Package for Social Science (SPSS) version 20. Data analysis and interpretation was done using descriptive statistics to determine frequencies and proportions. Logistic regression was employed to determine crude and adjusted odds ratios. A description with figures and tables was used for interpretation and displaying of data. At 95% confidence interval (CI), *p<*0.05 was considered statistically significant in all tests.

Ethical clearance was obtained from the ethical review committee of School of Pharmacy, Addis Ababa University and permission was obtained from the medical director and department of Internal Medicine, School of Medicine of ACSH to access patients’ medical records. The names of patients were replaced with codes to assure confidentiality. Only patients’ medical record was used and we didn’t interview patients; informed consent was not obtained from the patients to review their medical record instead we have asked the consent form the medical director and head department of internal medicine in accessing the patient medical record and the IRB waived the requirement for informed consent.

Killip class is the index of heart failure severity in patients with ACS based on physical examination. It was considered as Killip I: with no clinical signs of heart failure; Killip II: with rales in the lungs, third heart sound (S3), and elevated jugular venous pressure; Killip III: with acute pulmonary edema and Killip IV: with cardiogenic shock or arterial hypotension (measured as systolic blood pressure < 90 mmHg), and evidence of peripheral vasoconstriction (oliguria, cyanosis, and diaphoresis).

STEMI was considered as an acute heart attack resulted when an area of plaque within a coronary artery ruptures and forms a blood clot, suddenly blocking the supply of blood to a part of the cardiac muscles and resulting oxygen deprivation which is diagnosed by diagnosed by a 12-lead ECG test and the presence elevated cardiac biomarkers. NSTEMI was defined as a condition in which patients have acute chest pain but do not have persistent ST-segment elevation in their ECG and UA was also considered as clinical presentation and electrocardiographic finding is consistent with NSTEMI; but there is no elevation of cardiac necrosis markers.

In-hospital mortality was recorded if patients die while they are in the hospital due to ACS, which is explained as the proportion of mortality from those of ACS admitted patients. Treatment outcome was recorded as patients’ death in hospital, discharged with improvement or referred for further intervention. Hypertension was considered as systolic blood pressure (SBP) ≥140 mmHg, diastolic blood pressure (DBP) ≥ 90 mmHg, or the use of antihypertensive agents; dyslipidemia was also defined as patients on lipid-lowering agents, presence of one or more of the following four lipid disorders at a low-density lipoprotein ≥ 100 mg/dL, triglyceride level ≥ 150 mg/dL, and high-density lipoprotein level < 40 mg/dL and total cholesterol of ≥ 200 mg/dL and diabetes were presence of any of the following; fasting plasma glucose level ≥ 126 mg/dL, random blood glucose level ≥ 200 mg/dL, or a history of diabetes or patient on medication. Patients were considered also discharged with improvement if they had stable vital signs, normal cardiac biomarkers and alleviation of signs and symptoms during discharge.

## Results

Concerning Sociodemographic and clinical characteristics, of the total 151 patient records, near to three-fourths of them were males (72.2%) and were residing in urban areas (74.2%). The mean age was 59.12 years ([SD [standard deviation] ± 12.98]. The average time between onsets of symptoms to hospital admission was 95.85 hours [SD ± 145.68].Five (3.3%) patients presented within one hour onset of symptoms and 43% were admitted within 13‒72 hours. The average duration of hospitalization was 10.73 days [SD ± 8.63].Under half (44.4%) were discharged within seven days, and 20.5% were discharged after two weeks. The mean SBP was 123.89 mmHg [SD ± 29.61]. Eight (5.3%) patients had SBP of < 90 mmHg and 33.1% had ≥ 140 mmHg. The mean DBP was 77.43 mmHg [SD ± 15.98]. Eight (5.3%) patients had DBP of < 60 mmHg and above one-quarter (27.8%) were with DBP of ≥ 90 mmHg (Table 1).

**Table 1:** Sociodemographic and clinical characteristics of acute coronary syndrome patients admitted to Ayder Comprehensive Specialized Hospital, Mekelle, Ethiopia from August 2013‒July 2018.

Variable		Frequency (N)	Percent (%)
Sex			
Male Female	109	72.2	
	42		27.8
Residency			
Urban	112		74.2
Rural	39		25.8
Age in years[mean ± SD] [59.12 ± 12.98]			
< 60	74		49.0
60‒69	42		27.8
≥ 70	35		23.2
Time of presentation in hours [mean ± SD] [95.85 ± 145.68]			
<1	5		3.3
1‒12	40		26.5
13‒72	65		43.0
>72	41		27.2
Duration of hospitalization (days)			
≤ 7	67		44.4
8‒14	53		35.1
≥ 15	31		20.5
SBP [mean ± SD] [123.89 ± 29.61]			
< 90	8		5.3
90‒119	62		41.0
120‒129	22		14.6
130‒139	9		6.0
≥140	50		33.1
DBP [mean ± SD] [77.43 ± 15.98]			
< 60	8		5.3
60‒69	28		18.5
70‒79	37		24.5
80‒89	36		23.9
≥ 90	42		27.8

DBP: Diastolic blood pressure; SBP: Systolic blood pressure; SD: Standard deviation

**Fig 1:**
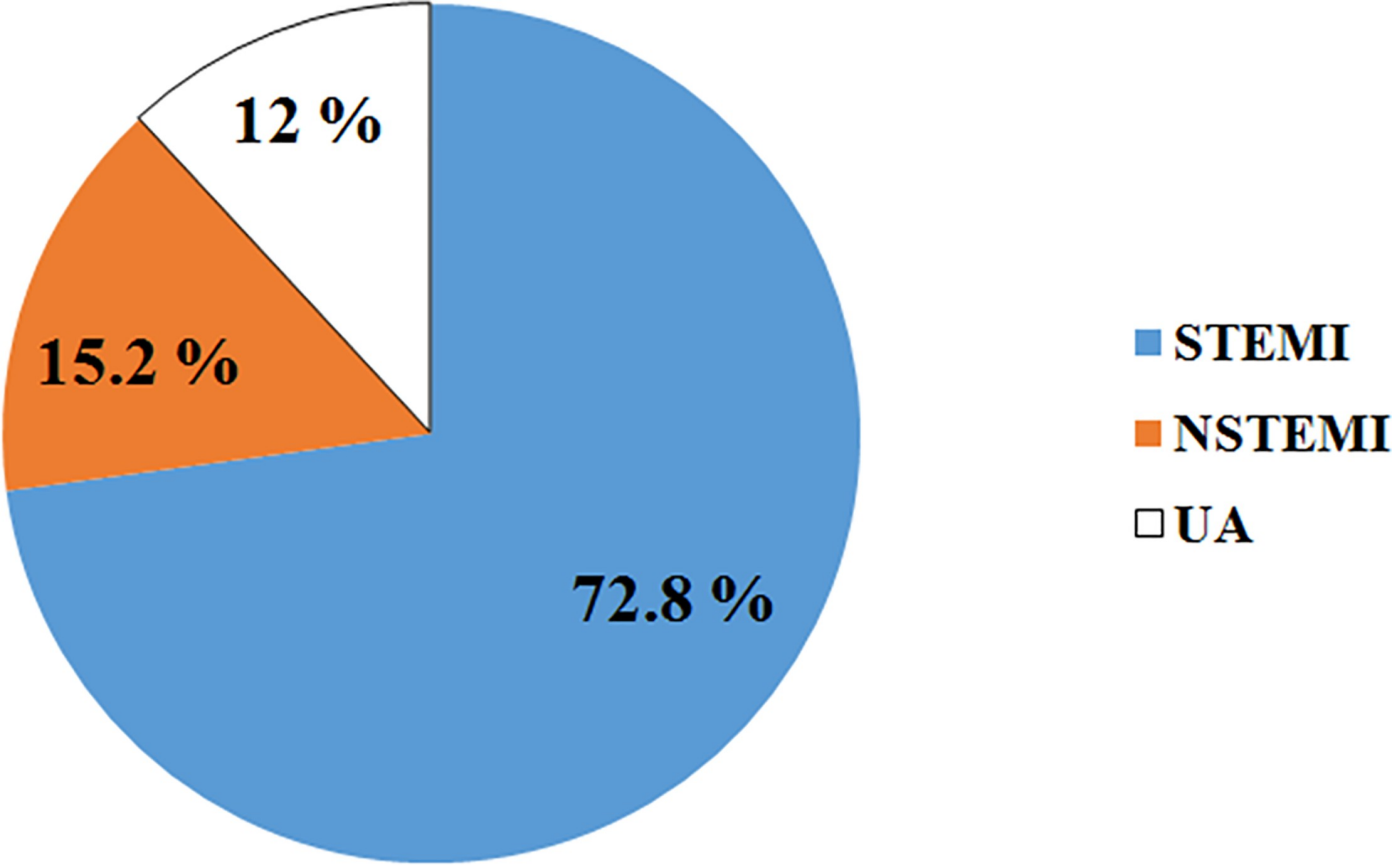
Final diagnosis of acute coronary syndrome patients admitted from August 2013‒July 2018.

As displayed in Fig 1, regarding to final diagnosis of ACS, majority of patients had STEMI in about 110 (72.8%), followed by NSTEMI 23 (15.2%) and then UA 18(12%) of patients (Fig 1).

As shown in Table 2, the frequently recorded patients’ symptoms on admission were chest pain (87.4%), followed by shortness of breath (33.8%). Out of 128 patients with evidence of killip class, 29.7% of them had killip class I and 20.3% class IV. Hypertension was the prominent risk factor of ACS (46.4%), followed by diabetes (34.4%) and then dyslipidemia (33.8%).

**Table 2:** Symptoms on admission, killip class and risk factors of acute coronary syndrome patients admitted to Ayder Comprehensive Specialized Hospital, Mekelle, Ethiopia from August 2013‒July 2018.

Variables	Frequency (N)	Percent (%)
Symptoms		
Chest pain	132	87.4
Shortness of breath	51	33.8
Nausea and vomiting	51	33.8
Cough	9	6.0
Sweating	31	20.5
Syncope	3	2.0
Killip class		
I	38	29.7
II	34	26.6
III	30	23.4
IV	26	20.3
Risk factors		
Dyslipidemia	51	33.8
Hypertension	70	46.4
Diabetes mellitus	52	34.4
Obesity	9	6.0
Family history of CAD	11	7.3
Previous MI	35	23.2
Exertional angina pectoris	16	10.6
Heart failure	9	6.0
Previous stroke	1	0.7
Smoking	18	11.9

CAD: Coronary artery disease; MI: Myocardial infraction

In case of laboratory and diagnostic investigations, as can be seen from the data in Table 3, from the recorded investigations, about one-fourth (25.9%) had elevated total cholesterol. Low density lipoprotein was higher than normal value in 40.6% of the patients. About 31.1% had lower than normal value of high density lipoprotein level and 30.6% had elevated Triglyceride level. On echocardiographic investigation, 29.8% had left ventricular ejection fraction (LVEF) ≤ 30%. Serum creatinine was raised in 28.9%. Elevated level of creatinine kinase-myocardial band and serum troponin was recorded in 49.4% and 83.2% of them, respectively.

**Table 3:** Profile of laboratory findings and diagnostic investigations of acute coronary syndrome patients admitted to Ayder Comprehensive Specialized Hospital, Mekelle, Ethiopia from August 2013‒July 2018.

Measured laboratory tests and diagnostic tools	Values	Frequency(N)	Percent (%)
Total cholesterol measured		104	68.9
	< 200	77	74.1
	≥ 200	27	25.9
LDL		32	21.2
	< 100	19	59.4
	≥ 100	13	40.6
HDL		47	31.1
	< 40	34	72.3
	≥ 40	13	27.7
TGs		98	64.9
	<150	68	69.4
	≥150	30	30.6
LVEF		124	82.1
	≤ 30	37	29.8
	> 30	87	70.2
SCr		149	98.7
	Normal	106	71.1
	Elevated	43	28.9
CK‒MB		79	52.3
	Normal	40	50.6
	Elevated	39	49.4
Troponin		137	90.7
	Normal	23	16.8
	Elevated	114	83.2

CK-MB: Creatinine kinase-myocardial band; HDL: High density lipoprotein; LDL: Low density lipoprotein; LVEF: Left ventricular ejection fraction; SCr: Serum creatinine; TGs: Triglycerides

Regarding to in-hospital management, in 27.1% patients’ cardiac catheterization was performed. Streptokinase was administered in seven (6.3%) patients with STEMI. Primary percutaneous coronary intervention (PCI) was done in six (3.9%) patients with STEMI. Loading dose of aspirin and clopidogrel were administered in the emergency setting in about 63.8% and 62.8% of patients, respectively. From those of the eligible patients, heparin derivatives were started in above three-fourths (78.1%)of them during hospitalization, while only one patient was initiated enoxaparin and the other patients were on unfractionated heparin. About 86.9% patients were treated using beta-blockers and all of those patients took metoprolol. Angiotensin converting enzyme inhibitors/angiotensin receptor blockers (ACEI/ARBs) were commenced in about 84.1% patients, where almost all were on enalapril and only two patients started with lisinopril. Nitrates were administered in 18.6% of patents, in which 14% were initiated with sublingual nitroglycerin, 3.9% isosorbide di-nitrate and one patient was started isosorbide mononitrate. About 84.1% patients took statins, of which 3.3% of them administered with simvastatin, while the other patients took high intensity dose of atorvastatin. Analgesic therapy of morphine was indicated in 11.9% and pethidine in 21.9% patients (Table 4).

**Table 4:** In-hospital management delivered for acute coronary syndrome patients admitted to Ayder Comprehensive Specialized Hospital, Mekelle, Ethiopia from August 2013‒July 2018.

Management			Final type of ACS			
			STEMI Frequency (%)	NSTEMI Frequency (%)	UA Frequency (%)	Total Frequency (%)
Catheterization			38 (34.5)	2 (8.7)	1(5.6)	41 (27.1)
Streptokinase			7 (6.3)	0 (0)	0 (0)	7 (6.3)
PCI			6 (5.5)	0 (0)	0 (0)	6 (3.9)
Aspirin						
		LD	68 (62.4)	14 (61.0)	13 (76.5)	95 (63.8)
		MD	109 (100)	22 (100)	18 (100)	149 (100)
Clopidogrel						
	LD		66 (44.6)	13 (59.1)	14 (82.4)	93 (62.8)
	MD		109 (99.1)	21 (100)	17 (100)	147 (99.3)
Heparins			77 (77.8)	21 (91.3)	9 (60.0)	107 (78.1)
Beta-blocker			88 (87.1)	16 (80.0)	15 (88.2)	119 (86.9)
ACEI/ARB			77 (85.6)	15 (78.9)	14 (82.4)	106 (84.1)
Nitrate			21 (22.8)	0 (0)	3 (16.7)	24 (18.6)
Statin			97 (74.1)	21(16.0)	13 (9.9)	131(84.1)
CCB			4 (36.4)	0 (0)	2 (11.1)	6 (3.9)
Morphine			15 (13.6)	1 (4.3)	2 (11.1)	18 (11.9)
Pethidine			22 (20.0)	4 (17.4)	7 (38.9)	33 (21.9)

ACEI/ARB: Angiotensin converting enzyme inhibitor/Angiotensin receptor blocker; ACS: Acute coronary syndrome; CCB: Calcium channel blocker; LD: Loading dose; MD: Maintenance dose; NSTEMI: Non-ST-elevation myocardial infraction; PCI: Percutaneous coronary intervention; STEMI: ST-elevation myocardial infraction; UA: Unstable angina

**Fig 2:**
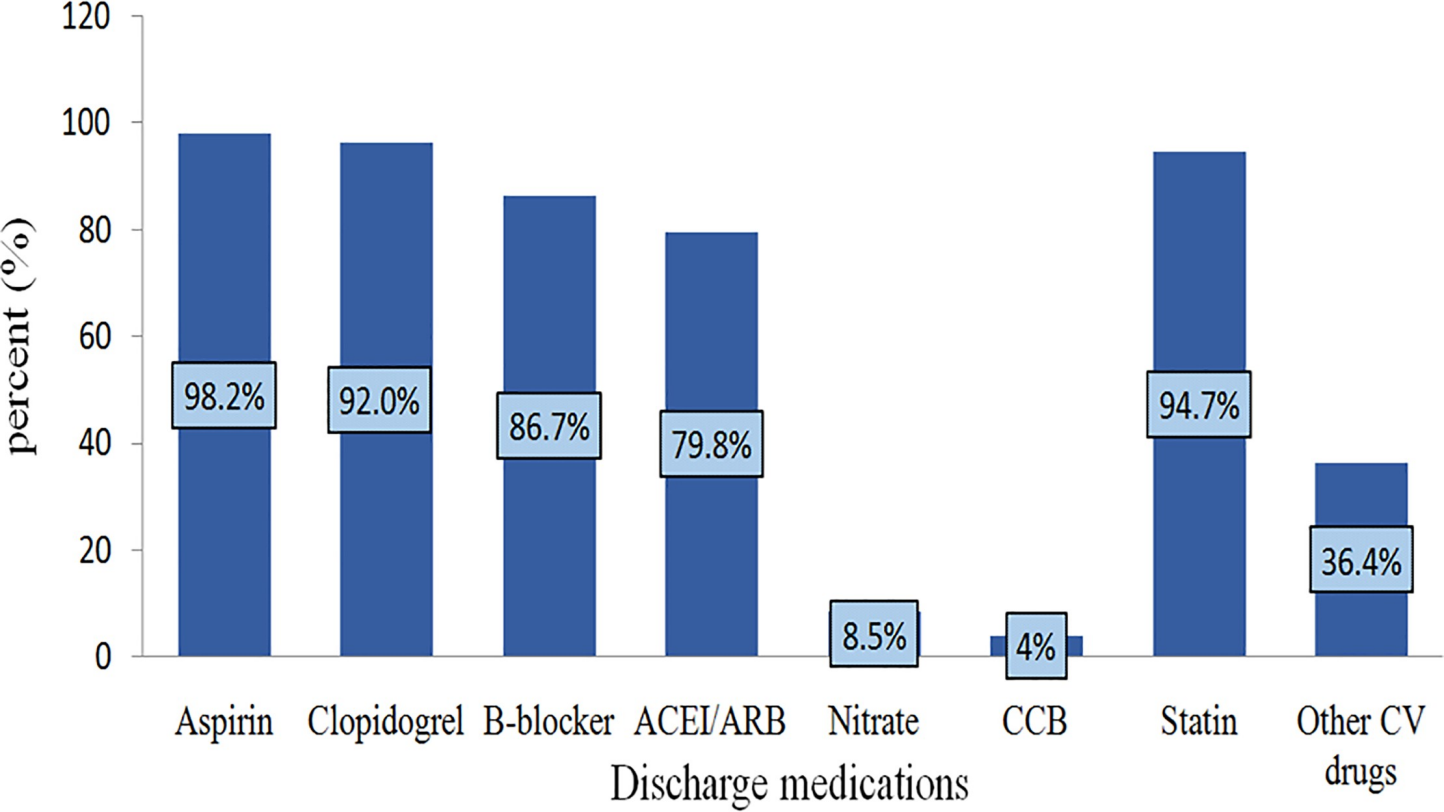
Discharge medications of acute coronary syndrome patients admitted from August 2013‒July 2018.

Regarding discharge medications, aspirin was prescribed in nearly all of the patients (98.2%). About 94.7% patients were discharged with statin and clopidogrel was prescribed in 92.0%. Beta-blocker and ACEI/ARBs were documented in 86.6% and 79.8% patients respectively. About 36.4% of patients were discharged with other cardiovascular drugs and less than 10% of the patients were discharged with nitrates (8.5%) (Fig 2).

With regard to treatment outcome and in-hospital complications, of the recorded hospitalization outcomes, 71.5% discharged with improvement. In-hospital mortality was recorded among 24.5%, where 67.5% of them had diagnosis of STEMI. Very few (3.9%) patients were referred for surgical intervention of coronary artery bypass grafting (CABG). The most common ACS-related complication was heart failure which was observed in about 42.4% of the patients. Subsequently, cardiogenic shock was recorded in 29.8% of patients. About 14.6% of patients developed myocardial re-infraction, and 11.3% had major arrhythmia. About 9.3% of patients had also neurologic complication of stroke and major bleeding episode was observed in 2.6% (Table 5).

**Table 5:** In-hospital outcome and complications of acute coronary syndrome patients admitted to Ayder Comprehensive Specialized Hospital, Mekelle, Ethiopia from August 2013‒July 2018.

		Final type of ACS			
Complications and in-hospital outcomes		STEMI	NSTEMI	UA	Total
		Frequency (%)	Frequency (%)	Frequency (%)	Frequency (%)
Complications					
	Heart failure	50 (78.1)	10 (15.6)	4 (6.3)	64 (42.4)
	Myocardial re-infraction	20 (90.9)	1 (4.5)	1 (4.5)	22 (14.6)
	Major arrhythmia	14 (82.3)	1 (5.9)	2 (11.7)	17 (11.3)
	Stroke	10 (71.4)	2 (14.2)	2 (14.2)	14 (9.3)
	Major bleeding episode	3 (75.0)	0 (0)	1 (25)	4 (2.6)
	Cardiogenic shock	35 (77.8)	8 (17.8)	2 (4.4)	45 (29.8)
Out comes					
	Death	25 (67.5)	9 (24.3)	3 (8.1)	37 (24.5)
	Discharged improved	81 (75.0)	14 (13)	13 (12)	108 (71.5)
	Referred for CABG	5 (83.3)	0 (0)	1 (16.6)	6 (3.9)

ACS: Acute coronary syndrome; CABG: Coronary arteries bypass grafting; NSTEMI: Non-ST-elevation myocardial infraction; STEMI: ST-elevation myocardial infraction; UA: Unstable angina

As indicated in Table 6, binary logistic regression was employed to determine predictors of in-hospital mortality in each of the following factors; Sex, age, residency, duration of symptom onset to admission, duration of hospitalization, symptoms, blood pressure, cardiovascular comorbidities, risk factors, final type of ACS, klipp class, serum myocardial necrosis markers, serum creatinine, lipid profiles, left ventricular ejection fraction, electrocardiography, cardiac catheterization, reperfusion therapy and type of medication initiated. The factors were assumed statistically significant at *p*<0.05.

**Table 6:** Contributing factors for in-hospital morality of acute coronary syndrome patients admitted to Ayder Comprehensive Specialized Hospital, Mekelle, Ethiopia from August 2013‒July 2018.

Variable		Death		COR [95% CI]	*p*-value	AOR [95% CI]	*p*-value
		Yes, n (%)	No, n (%)				
Age	< 60	12 (16.2)	62 (83.8)	0.371 [0.146–0.942]	0.037*	0.185 [0.025–1.358]	0.097
	60–69	13 (31)	29 (69)	0.859 [0.330–2.236]	0.756	0.729 [0.108–4.926]	0.746
	≥ 70	12 (34.3)	23 (65.7)	1.000		1.000	
Clopidogrel LD in emergency	Yes	18 (19.4)	75 (80.6)	1.000		1.000	
	No	19 (34.5)	36 (65.5)	2.199 [1.031–4.690]	0.041*	1.308 [0.263–6.510]	0.743
B-blocker	Yes	18 (15.1)	101 (84.9)	1.000			
	No	19 (59.4)	13 (40.6)	8.201[3.451–19.487]	< 0.0001*	8.722 [1.560–48.776]	0.014*
ACEIs/ARBs	Yes	14 (13.2)	92 (86.8)	1.000		1.000	
	No	23 (51.1)	22 (48.9)	6.870 [3.053–15.459]	< 0.0001*	3.365 [0.610–18.574]	0.164
Heart failure	Yes	22 (34.4)	42 (65.6)	1.000		1.000	
	No	15 (17.2)	72 (82.8)	0.398 [0.186–0.849]	0.017*	0.911 [0.185–4.500]	0.909
Cardiogenic shock	Yes	31 (68.9)	14 (31.1)	1.000		1.000	
	No	6 (5.7)	100 (94.3)	0.027 [0.010–0.076]	< 0.0001*	0.055 [0.011–0.290]	0.001*
Klipp class	Class I,II	9 (12.9)	61 (87.1)	1.000		1.000	
	Class II,III	23 (39.7)	35 (60.3)	0.225[0.094–0.539]	0.001*	0.355 [0.069–1.832]	0.216
LVEF	≤ 30	18 (48.6)	19 (51.4)	1.000		1.000	
	> 30	10 (11.5)	77 (88.5)	0.137 [0.055–0.345]	< 0.0001*	0.176 [0.036–0.865]	0.032*

*Statistically significance; *p*<0.05

ACEI/ARBs: Angiotensin converting enzyme inhibitor/angiotensin receptor blockers, AOR: Adjusted odds ratio, CI: Confidence interval, COR: Crude odds ratio, HF: Heart failure, LD: Loading dose, LVEF: Left ventricular ejection fraction

Based on the multivariable regression analysis, initiation of beta-blockers with in the first day of hospitalization had 8.722 times more reduction of in-hospital mortality (AOR(adjusted odds ratio) = 8.722; 95% CI: 1.560–48.776). Patients who had in-hospital complication of cardiogenic shock were 18.181 times more likely to die (AOR = 0.055; 95% CI: 0.011–0.290). Patients who had reduced LVEF (≤ 30%) were 5.681 times more likely to die in-hospital than patients who had LVEF of > 30% (AOR = 0.176; 95% CI: 0.036–0.865).

**Fig 3:**
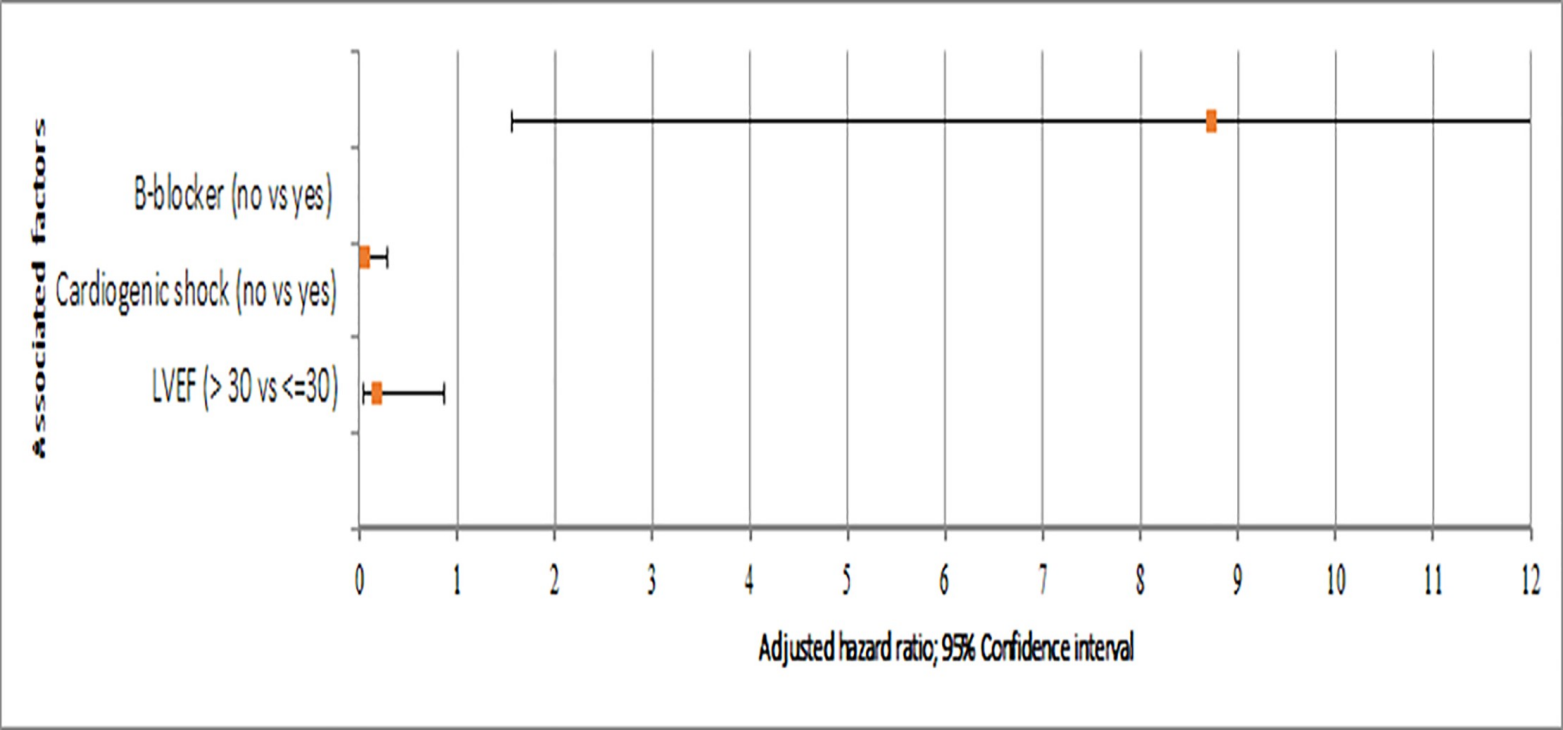
Predictors for in-hospital mortality of acute coronary syndrome patients from August 2013‒July 2018.

As described in Fig 3, in multivariable regressions; patients who did not use B-blockers with in the first day of hospitalization, development of in-hospital complication of cardiogenic shock and patients who had reduced LVEF (≤ 30%) were more likely to die in-hospital (Fig 3).

## Discussion

In this study, the most common risk factor for ACS was hypertension (46.4%). Similar finding was claimed by other studies too; hypertension was the most frequent risk factor in India (48.4%) [22], Nepal (68%) [23], Greece (58.8%) [24]and Senegal (46%) [25]. Plausible explanation could be due to hypertensive patients might have poor knowledge and attitude on the prevention of cardiovascular complications; such as adhering life style adjustment, having appropriate follow up and adhering to their medications accordingly [26].

In the current study, thrombolytic medications were administered in about 6.3% of patients with STEMI. This finding illustrated that very few of the patients with STEMI have used thrombolytic therapy. Based on European society of cardiology (ESC) 2017 guideline recommendation, for STEMI type of patients admitted within 12 hours of symptom onset, thrombolytic should be initiated within 30 minutes of first medical contact (FMC); if no contraindication [27]. This is due to, the presence of complete blockage of the coronary artery in STEMI patients. Therefore, thrombolytic should be initiated immediately within the recommended time of presentation [28]. In the present study, thrombolytic administration was not consistent with the recommendation of clinical guidelines. This might be due to time delay between symptom onset and admission, since the average time of admission was 95.85 hours (4 days) [SD ± 145.68]. The time delay could be due to, patients inadequate awareness about the symptoms of ACS, which was described in a study from Egypt [29]. The other possible reason for the time delay could be due to, problem in transportation of the patients to health institution, in which 25.8% of the patients were rural residents. Another plausible reason could be lack of adequate accessibility and affordability of the medication in relation to the socio-economic status of the patients.

Our study revealed a lower percentage of thrombolytic use compared to studies from Kenya 79.5% [30], South Africa 18% [31], sub‒Saharan countries 44.4% [32] and Iran 46.3% [33]. This could be due to better availability and accessibility of thrombolytic medications in Kenya, South Africa and similarly in Iran too.

In the present study, very few (3.9%) patients were enrolled for PCI intervention. Nevertheless PCI is the most effective reperfusion therapy for symptomatic ACS patients admitted within 90 hours. This therapy is very important especially for sever forms ACS [34]. Even though the service is started in the study setting; in this study, limited patients have benefited from it. In this setting, it was done mostly for stable patients rather than during acute phase of admission. This might be due to problem of PCI service accessibility and affordability. Another reason could also be due to delayed admission time to the hospital, where the mean time of presentation was around four days. In contrary, the finding of the present study was lower than reports from Iran 17.3% [33], South Africa 53%(31), Kenya 38% [35], Greece 27.0% [36] and Ethiopia 65.3% [37]. Possibly, the inconsistency is due to wide accessibility of PCI service in these areas.

In our study, in the emergency setting loading dose of aspirin and clopidogrel were used in 63.8% and 62.8%, respectively. Dual antiplatelet therapy should be started for ACS patients upon admission to the emergency setting, for prevention of cardiovascular complications and mortality [27]. In the current study, the documented dual antiplatelet therapy was not consistent with the guidelines’ recommendation. Possibly, the reasons could be that some patients might take the medication in private hospitals or other health institutions; and patients might take the drug but it may not be documented. Chest pain was not manifested in 12.6% of them; this may also resulted in delayed initiation of these dual anti-platelet therapies, since chest pain is cardinal symptom of ACS [28]. Comparably, the present finding was lower than studies from Greece [38], aspirin and clopidogrel were used in 97% and 93% patients and in Brazil [39], aspirin and clopidogrel were prescribed in 95% and 88.7% patients, respectively.

In the present study, in-hospital mortality due to ACS was 24.5%, where majorities (67.5%) of them were due to STEMI, followed by NSTEMI (24.3%) and then UA (8.1%). This study showed that ACS accounted considerably high proportion of in-hospital mortality. This might be due to the fact that majority of the patients had STEMI type of ACS which increases risk of mortality that is due to complete blockage of the coronary artery. This was described similarly by studies in Poland [40], GRACE [41] and international registry of ACS in transitional countries [42]. Other possible reason could be due to delayed time of presentation to hospital setting. The finding of the current study was considerably comparable with study conducted in Ethiopia, Tikur Anbessa Specialized Hospital which was 29.4% [12]. This study was also relatively consistent with another study from Ethiopia, Tikur Anbessa Specialized Hospital conducted from January 1, 2012 to December 31,2014was 27.4% [13]. Moreover, our study was also relatively similar with studies from; Nigeria 21.4% [43], Senegal 21% [25], India 18.4% [44] and Kenya 17% [45]. Inadequate use of reperfusion therapy, similarly explained by both of the studies in Ethiopia and Senegal, might be a reason for the mortality rate. In the study setting, the delayed initiation of invasive diagnostic and therapeutic options, coronary angiography started on February 2016 and PCI on April 2016, might explain the mortality rate. Similarly, another study from south east Asian setting reported that unavailability of revascularization therapy was associated with high mortality rate (HR; 2.38, *p* = 0.005) [46].

However, our finding was higher than studies from India 3.9% [22], Czech republic 4.2% [47], Brazil 9.4% [48], Pakistan 12.2% [49], Kenya 9.4% [30] and Sub-Saharan African countries 10% [10]. On one hand, it might be due to sample size variation; in this study only 151 patients were enrolled where as the sample size in Brazil, India and Pakistan was larger. And on the other hand in those areas better reperfusion therapy (PCI, thrombolytic and CABG) was widely available.

In case of predictors for in-hospital mortality, in this study, multivariate logistic regression were used to reduce the effect of confounding factors; reduced LVEF of ≤ 30%, presence of cardiogenic shock as complication of cardiogenic shock and non-use of B-blocker therapy were found to be significantly associated with in-hospital mortality of ACS.

In our study, in-hospital initiation of beta-blockers within the first day of admission had 8.722 times more preventive effect for in-hospital mortality (95% CI: 1.560–48.776). Evidence-based in-hospital initiation of beta-blockers in patients with ACS reduces the risk of morbidity and mortality. Beta-blockers prevent cardiac remodeling, re-infraction and post-MI heart failure complication in hemodynamically stable patients [50]. In this study, the use of beta-blocker was found to be sub-optimal. Initiation of beta-blocker was omitted during the first 24 hours of the patients’ admission. The reason might be due to presence of patients who are relatively contraindicated, which could be hemodynamically unstable initially but subsequently, these patients improved and were candidate for B-blocker initiation. Hence, initiation of beta-blocker for these patients was missed upon the patient progress improvement and hemodynamic stability, which could be started by low dose and will be escalated based on the patient’s condition. It could be also due to; medication supply interruption. The other possible reason might be also, the patient took the medication, but it might lack documentation. This finding was in line with retrospective study from Brazil, where early initiation of beta-blocker had reduced in-hospital mortality by 8.12 times (95% CI:1.53–14.56) [51]. Similarly, from Switzerland Erne and his co-authors claimed that starting of beta-blockers had preventive effect of in-hospital mortality by 2.174 times (AOR = 0.46, 95% CI:0.37–0.57) [52]. The present study was also supported by retrospective study from Russia, initiation of beta-blockers had reduced in-hospital mortality (*p*<0.0001) [53].

LVEF determines functional capability of the left ventricle in pumping or the percentage of blood pumped per single contraction of the left ventricle [54]. In this study, patients who had LVEF of ≤ 30% were 5.681 times more likely to die in-hospital than LVEF of > 30% (AOR = 0.176; 95% CI: 0.036–0.865). In the current study, it might be the case that heart failure was recorded in about 34.4% of the deaths and about 39.7% of them had also advanced klipp class III and IV. The risk of mortality could be exacerbated in the presence of heart failure with advanced klipp class that will result in reduced left ventricular ejection fraction [55]. Furthermore, the possible explanation could be that non-use of timely management of invasive reperfusion therapy. Likely, studies from Iran claimed that, patients who had LVEF ≤ 30% were 4.83 times were more likely to die in-hospital (95% CI: 2.72‒8.56) [56]. Another similar study by Kurtul and Ozturk investigated that patients who had LVEF less than 40% were 2.381 times more likely to die (*P* = 0.003) [57]. Comparably, in Romania Cretu and his co-authors reported that LVEF < 35% was associated with higher(3.8 times) in-hospital mortality (95%CI: 2.6–5.4) [58]. Similarly, Perelshtein *et al* showed patients with evidence of LVEF < 30% were 4.49 times more likely to die than who had LVEF of > 50% (95% CI:3.57–5.61) [59].

In the present study, in-hospital complication of cardiogenic shock was found to be another independent predictor of in-hospital mortality. We found that patients who had in-hospital cardiogenic shock as complications were 18.181 times more likely to die in-hospital (AOR = 0.055; 95%CI: 0.011–0.290). As explained by Kataja and Harjola, in patients with cardiogenic shock, there will be a significant reduction in cardiac output after myocardial insufficiency. Subsequently, the reduction in blood pressure further activates the sympathetic activity and systemic vascular resistance. Consequently, upon continuation of this imbalance there will be low perfusion and end organ damage, then this will lead to death [60]. Patients with cardiogenic shock in ACS should be treated with reperfusion therapy; supportive fluids and ionotropic agents [61]. The above finding could be due to inadequate use of reperfusion therapy [62]. However, a retrospective study conducted in Oman reported that out of 63 ACS patients having cardiogenic shock, the in-hospital mortality was 52.4%, even though 93.6% of them underwent PCI [63]. This difference could be due to sample size variation and timing of the therapeutic intervention.

Our finding was in agreement with a study done by Dziewierz *et al*, where patients with cardiogenic shock were 7.39 times more likely to die in-hospital (95% CI:4.50–12.11) [64]. Similar finding from Afghanistan claimed that, of 351 patients with cardiogenic shock, the in-hospital mortality was reported about 44.7% [62].

Certain limitations such as single centered, retrospective cross-sectional study design and small sample size might have affected this study.

## Conclusion

In this study, in-hospital mortality of ACS was found to be high. Non-use of beta-blockers within the first day of hospitalization, development of cardiogenic shock as complication and LVEF of ≤ 30% were found to be independent predictors of in-hospital mortality. This study also revealed that few patients were managed by PCI and thrombolytic therapy, even though limited services are available. Prospective, multicenter study and with greater sample size should be used in future studies for generalization and representativeness of the whole population. Evidence-based and timely management should be implemented in the study setting. Health-related policy makers should work on the wide accessibility for the advanced therapies of PCI and thrombolytic in accordance with the patients socio-economic status, for prevention of death, ACS related complications and to improve patients quality of life.

## Supporting information

S1 File (DOC)

## Abbreviations

ACEI/ARBs: Angiotensin Converting Enzyme Inhibitor/Angiotensin Receptor Blockers
ACS: Acute Coronary Syndrome
ACSH: Ayder Comprehensive Specialized Hospital
AHA: American Heart Association
AMI: Acute Myocardial Infraction
AOR: Adjusted Odds Ratio
CABG: Coronary Artery Bypass Grafting
CAD: Coronary Artery Disease
CVD: Cardiovascular Disease
CV: Cardiovascular
DBP: Diastolic Blood Pressure
ESC: European Society of Cardiology
FMC: First Medical Contact
GRACE: Global Registry of Acute Coronary Events
HMIS: Health Management Information System
LBBB: Left Bundle Branch Block
LVEF: Left Ventricular Ejection Fraction
NSTEMI: Non-ST-Elevation Myocardial Infraction
PCI: Percutaneous Coronary Intervention
SBP: Systolic Blood Pressure
SD: Standard Deviation
STEMI: ST-Elevation Myocardial Infraction
UA: Unstable Angina
WHO: World Health Organization
