# Correction: Treatment outcome of acute coronary syndrome patients admitted to Ayder Comprehensive Specialized Hospital, Mekelle, Ethiopia; A retrospective cross-sectional study

**DOI:** 10.1371/journal.pone.0247961

**Published:** 2021-02-25

**Authors:** Desilu Mahari Desta, Teshome Nedi, Abraha Hailu, Tesfay Mehari Atey, Afewerki Gebremeskel Tsadik, Solomon Weldegebreal Asgedom, Gebremicheal Gebereslassie Kasahun, Eskinder Ayalew

The sixth author’s name is spelled incorrectly. The correct name is: Solomon Weldegebreal Asgedom.
