# Supplementary material for: Treatment outcome of acute coronary syndrome patients admitted to Ayder Comprehensive Specialized Hospital, Mekelle, Ethiopia; A retrospective cross-sectional study
Source: PLoS One. 2020 Feb 13;15(2):e0228953. doi: 10.1371/journal.pone.0228953 (PMC7018065; doi:10.1371/journal.pone.0228953)
Supplement: S1 File — (DOC) [file pone.0228953.s001.doc]

**S1 file**

**Data abstraction tool**

1. Patient’s detail and admission information

1.1. Medical record number:___________

1.2. Age:_________

1.3. Sex: male
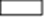
, female
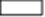


1.4. Residency: urban
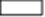
, rural
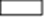


1.5. Date of admission:____________

1.6. Date of discharge/death: ____________

1.7. Time of death after admission:________

- 1. Time between symptom onset and hospital admission:_______
  2. Length of hospital stay:­­­­_________

1.10. Compliant/symptom on admission:

Chest pain
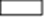
 nausea and vomiting
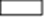


Shortness of breath
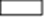
 others specify____________

1.11. P/E on admission:

BMI:_______

BP: ________

HR:_________

RR:_________

1. Medical history and risk factors

| Medical history/risk factor | Yes | No | Unknown |
| --- | --- | --- | --- |
| - 1. Dyslipidemia |  |  |  |
| - 1. Hypertension |  |  |  |
| - 1. Diabetes mellitus |  |  |  |
| - 1. Obesity |  |  |  |
| - 1. Family history of CAD |  |  |  |
| - 1. Previous MI |  |  |  |
| - 1. Exertional angina pectoris |  |  |  |
| - 1. Heart failure |  |  |  |
| - 1. Previous stroke or TIA |  |  |  |
| - 1. Previous smoker |  |  |  |
| - 1. Current smoker |  |  |  |
| - 1. Never smoked |  |  |  |

1. Final type of ACS diagnosis
   1. STEMI
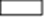

   2. NSTEMI
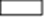

   3. UA
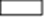

2. Investigations and assessments during admission
   1. Killip Class ___________
   2. Continuous ECG monitoring
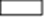
YES
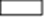
 NO
   3. Serum CK-MB measured
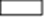
YES
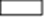
NO If YES peak value_____
   4. Serum Troponins measured
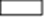
 YES
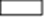
 NO If YES peak value_____
   5. Serum Creatinine measured
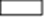
YES
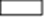
 NO If YES peak value_____
   6. Serum Lipid profiles measured during admission
       4.6.1.Total cholesterol
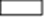
YES
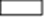
 NO ,If YES value_____mg/dL
       4.6.2.LDL cholesterol
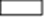
YES
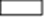
NO , If YES value_____mg/dL
       4.6.3.HDL cholesterol
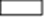
YES
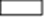
NO , If YES value_____mg/dL
       4.6.4.Triglyceride
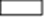
YES
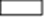
NO , If YES value_____mg/dL
   7. Echocardiogram performed
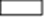
YES
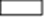
NO, If yes LVEF_______%
   8. Cardiac catheterization performed
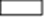
YES
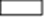
NO

5. Reperfusion therapy/ revascularization

5.1. Thrombolytic therapy given
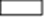
YES
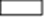
NO

if yes which drug and dose
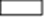
Alteplase
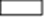
Reteplase


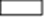
 Streptokinase
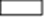
Tenecteplase

Other__________________

Tot. Dose__________mg

FMC to Needle Time ________________

If NO the reason why
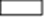
Given before arrival
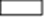
Primary PCI


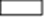
 Contraindication
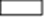
 Not indicated


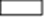
Patient refused
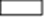
 Unknown


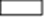
Patient cannot afford
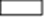
 Delayed admission time

5.2. PCI performed
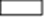
YES
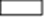
 NO

If yes the first procedure is
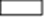
 Primary (Emergency)


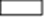
Rescue


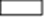
 Elective

If NO the reason why
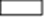
 Thrombolysis before arrival
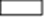
 Primary thrombolysis


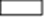
Contraindication
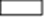
 not indicated


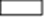
Patient refused
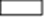
 Unknown


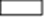
 Patient cannot afford
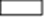
 Delayed admission time

5.3. CABG performed
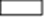
 YES
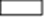
NO

6. In-hospital medical therapy initiated

| Drug name | YES | | NO | Started in Emergency department | | |
| --- | --- | --- | --- | --- | --- | --- |
|  | Loading Dose(mg) | Maintenance dose(maximum dose) (mg) |  | Yes | no | if no reason |
| 6.1. Aspirin |  |  |  |  |  |  |
| 6.2.Clopidogrel |  |  |  |  |  |  |
| 6.3. Anti-coagulant |  |  |  |  |  |  |
| 6.4. Beta Blocker |  |  |  |  |  |  |
| 6.5. Morphine |  |  |  |  |  |  |
| 6.6. Nitrates (SL/PO) |  |  |  |  |  |  |
| 6.7.ACEIs/ARBs |  |  |  |  |  |  |
| 6.8. Ca-channel blocker |  |  |  |  |  |  |
| 6.9. Statins |  |  |  |  |  |  |

7. Discharge Medications

| Drug name | Yes | No | If no reason |
| --- | --- | --- | --- |
| 7.1. Aspirin |  |  |  |
| 7.2. Clopidogrel |  |  |  |
| 7.3. Beta Blocker |  |  |  |
| 7.4. ACEIs/ARBs |  |  |  |
| 7.5 Nitrates (oral) |  |  |  |
| 7.6. Ca2+-channel blocker |  |  |  |
| 7.7. Statins |  |  |  |
| 7.8. Other cardiovascular Drug |  |  |  |

8. Major in-hospital complications and hospitalization outcomes

| In-hospital comorbidities and outcomes | Yes | No |
| --- | --- | --- |
| 8.1. CHF |  |  |
| 8.2. Myocardial re-infarction |  |  |
| 8.3. Major arrhythmia |  |  |
| 8.4. Stroke |  |  |
| 8.5. Major bleeding episode |  |  |
| 8.6. Cardiogenic shock |  |  |
| 8.7. Death |  |  |
| 8.8. Discharged Improved |  |  |
| 8.10. Referred |  |  |
